# Chromium on the Hands of Children After Playing in Playgrounds Built from Chromated Copper Arsenate (CCA)–Treated Wood

**DOI:** 10.1289/ehp.8521

**Published:** 2005-10-06

**Authors:** Camille Hamula, Zhongwen Wang, Hongquan Zhang, Elena Kwon, Xing-Fang Li, Stephan Gabos, X. Chris Le

**Affiliations:** 1 Environmental Health Sciences, Department of Public Health Sciences, University of Alberta, Edmonton, Alberta, Canada; 2 Health Surveillance Branch, Alberta Health and Wellness, Edmonton, Alberta, Canada

**Keywords:** CCA, children’s exposure, chromated copper arsenate, chromium, playgrounds, treated wood

## Abstract

Children’s exposure to arsenic and chromium from playground equipment constructed with chromated copper arsenate (CCA)–treated wood is a potential concern because of children’s hand-to-mouth activity. However, there exists no direct measure of Cr levels on the hands of children after playing in such playgrounds. In this study we measured both soluble and total Cr on the hands of 139 children playing in playgrounds, eight of which were constructed with CCA-treated wood and eight of which were not. Children’s age and duration of play were recorded. The hands of each child were washed after play with 150 mL deionized water, which was collected in a bag and subsequently underwent analysis of Cr and 20 other elements, using inductively coupled plasma mass spectrometry. Total average Cr on the hands of 63 children who played in CCA playgrounds was 1,112 ± 1,089 ng (median, 688; range 78–5,875). Total average Cr on the hands of 64 children who played in non-CCA playgrounds was 652 ± 586 ng (median, 492; range 61–3,377). The difference between the two groups is statistically significant (*p* < 0.01). Cr levels were highly correlated to both Cu (*r* = 0.672) and As (*r* = 0.736) levels in CCA playgrounds (*p* ≤ 0.01), but not non-CCA playgrounds (*r* = 0.252 and 0.486 for Cu and As, respectively). Principal-component analysis indicates that Cr, Cu, and As are more closely grouped together in CCA than in non-CCA playgrounds. These results suggest that the elevated levels of Cr and As on children’s hands are due to direct contact with CCA wood.

Chromated copper arsenate (CCA) has been widely used for > 60 years as a wood preservative to extend the lifetime of wood as a building material (Bull 2001; Nico et al. 2004). The wood is treated by impregnation with CCA under high pressure, resulting in deposition of copper, chromium, and arsenic oxides in the wood (Lebow et al. 2003). The most common formulation of CCA in North America is CCA-C, consisting of 18.5% copper oxide, 47.5% chromium oxide, and 34% arsenic oxide (Hingston et al. 2001). In both Canada and the United States, manufacturers of wood-treatment chemicals voluntarily agreed to cease use of CCA on lumber for nonindustrial purposes as of December 31, 2003 [Health Canada Pest Management Regulatory Agency (HCPMR) 2002; U.S. Environmental Protection Agency (EPA) 2002a). However, many residential structures built from CCA are still in place, including playground equipment and decks. In the United States, 14% of public playgrounds contain CCA-treated wood (U.S. EPA 2004). In Edmonton, Alberta, Canada, as of August 2003, 222 of 316 public playgrounds owned and operated by the city contained CCA-treated wood.

Because of the toxicity of Cr(III) and inorganic As species [Agency for Toxic Substances and Disease Registry (ATSDR) 2000; National Research Council 1999], children’s exposure to Cr or As from CCA-treated playground structures and decks is of potential health concern (Hemond and Solo-Gabrielle 2004). CCA component leaching increases with weathering of treated wood (Lebow et al. 2003, 2004a). Many studies have determined As and Cr in soil and sand, often with a primary objective of understanding the leaching of As and/or Cr into sand and soil (Balasoiu et al. 2001; Cooper et al. 2001; Hingston et al. 2001 for review; Lebow et al. 2004a; Stilwell and Gorny 1997; Townsend et al. 2003; Zagury et al. 2003). Previous analyses of risk and exposure, therefore, relied on indirect data, including the concentration of As and/or Cr in the soil and sand, projections of the amount of CCA material that ends up on children’s hands based on average child hand surface area, adherence factors for soil, and child behavioral patterns (U.S. EPA 2002b, 2003a, 2003b). No previous study has directly measured Cr levels on the hands of children after playing in CCA playgrounds. The purpose of this study is to fill this gap.

Recently, Kwon et al. (2004) quantified the amount of As on children’s hands after playing in playgrounds built from CCA-treated wood. The procedure involved taking hand-washing samples of 66 children from eight CCA playgrounds and 64 children from eight non-CCA playgrounds. After play, the hands of each child were rinsed with water and the amount of As in the rinsate was measured, thus allowing comparison between CCA and non-CCA playgrounds. Children from CCA playgrounds had a maximum of approximately 4 μg As on their hands, which is five times greater, on average, than in non-CCA playgrounds. To determine Cr on children’s hands, the samples from the Kwon et al. study (2004) were re-examined by undergoing an inductively coupled plasma mass spectrometry (ICPMS) analysis of 20 elements, including Cr, As, and Cu. The analysis provided the first direct measurements of Cr on children’s hands, eliminating the uncertainties associated with estimating the levels of Cr on children’s hands. The data allowed for correlation analyses of Cr, As, and Cu levels. Principal-component analysis (PCA) of the multi-element data from both CCA and non-CCA playgrounds may provide further information on the characteristic grouping of As, Cr, and Cu, as well as the dislodging of these elements from CCA-treated wood.

## Materials and Methods

### Playground selection and sample collection.

Playgrounds were designated as either CCA or non-CCA, with CCA signifying those either totally or partially constructed from CCA-treated wood. A total of 16 playgrounds from the city of Edmonton—eight CCA and eight non-CCA—were used in the study and were chosen to represent the distribution of site characteristics found across the city. Both CCA and non-CCA playgrounds were similar in age, location, and manufacturer. The study protocol was reviewed and approved by the University of Alberta Health Research Ethics Board.

The samples used were the same as those used in Kwon et al. (2004). Selected sites were sampled in random order in August 2003. As children arrived at a playground, parental permission was obtained in the form of written consent to allow the children’s participation in the study. On average, seven to nine children were sampled at each site. Upon completing their play, each child provided hand-washing samples by washing hands for 1 min in Ziploc bags (18 × 20 cm; Johnson and Son Ltd., Brantford, ON, Canada) filled with 150 mL deionized water. A blank sample of deionized water was also prepared for each site and except for hand-washing, subjected to the same procedures, including transportation, as the other samples. The age and length of play time of each child was recorded, and the hand-washing samples were poured into polystyrene bottles. Each bag was rinsed with an additional 80 mL water and combined with the corresponding 150 mL rinsate, yielding a 230-mL sample from each child. Samples were then filtered with Whatman glass microfiber 1.2-μm filters (Whatman International Ltd., Maidstone, UK). The filtrate (soluble fraction) and the residual sand collected on the filters were separately stored at 4°C until used. For a more detailed description of playground selection and sample collection, please consult Kwon et al. (2004). Samples from 63 children who played in CCA playgrounds and 64 children who played in non-CCA playgrounds were available for this study.

### Determination of soluble chromium and 20 elements in the soluble fraction of the hand-washing samples.

Ten milliliters of each filtered sample was acidified to a final concentration of 1% nitric acid (aq). Concentrations of total Cr were determined in nanograms per milliliter (parts per billion) for each sample. Cr concentration was multiplied by the volume of each sample (230 mL) to yield the total amount of Cr (nanograms) on children’s hands.

The samples were analyzed using an ICPMS (6100DRC Plus; Perkin-Elmer/Sciex, Concord, Ontario, Canada). A multielement analysis was carried out for each sample for the following elements: As, beryllium, barium, bismuth, cadmium, cobalt, Cr, Cu, iron, gallium, indium, magnesium, manganese, nickel, lead, rubidium, selenium, strontium, thallium, vanadium, and zinc. The liquid was introduced via a Meinhard nebulizer coupled with a cyclonic spray chamber. An ASX-500 autosampler (CETAC Technologies Inc., Omaha, NE, USA) was used. The RF power was 1.3 kW. Argon gas flow rate was 15 L/min (plasma gas), 0.8 L/min (nebulizer gas), and 1.5 L/min (auxiliary gas). Calibration of the ICPMS was carried out using six Cr concentrations (0, 5, 10, 15, 20, and 25 ppb) at the beginning of each run, as well as after every 10 samples with 15 ppb Cr. National Institute of Standards and Technology (NIST) Standard Reference Material (SRM) 1640, Trace Elements in Natural Water (NIST, Gaithersberg, MD, USA), was analyzed once during each run as a quality control. This SRM was diluted 2-fold before analysis. The average measured value of Cr in the SRM was 34.2 ± 4.1 μg/L from the repeat analyses over 3 days, which is in agreement with the certified SRM value of 38.6 ± 1.6 μg/L.

### Determination of chromium and 20 elements in the insoluble fraction of hand-washing samples.

Because the hand-washings contained residual particles from the children’s hands, the amount of residue collected on the filters and the concentrations of Cr in the residue were determined separately from those in the solutions of the hand-washings. Hand-washing samples were filtered using Whatman glass microfiber filters with 1.2-μm pores. The filter was then dried at 140ºC and weighed, to determine the exact amount of sand collected on the children’s hands.

The sand, along with the filter, was digested with a mixture of concentrated HNO_3_/perchloric acid/hydrofluoric acid (1:1:1 volume ratio). Initially, the mixture was heated to 40–60ºC for 1 hr, followed by heating at 100ºC to completely dissolve all solid material. It was then boiled for 1 hr to evaporate the acids to almost dryness, then was redissolved in 1% HNO_3_ in preparation for ICPMS analysis as described previously (Kwon et al. 2004). Total Cr concentrations in the sand were then determined via a multi-element analysis using an ELAN 6000 ICPMS (Perkin-Elmer/Sciex). The same 21 elements were analyzed as for the hand-washing solutions.

### Playground sand/soil samples.

Three composite sand/soil samples were taken from each playground site on the same day as the hand-washing samples. Extensive sand/soil sampling was done in playgrounds G and R (24 samples collected in each). Sampling was carried out as previously described (Kwon et al. 2004).

### Determination of arsenic, chromium, and copper in sand and soil samples.

The levels of As, Cr, and Cu in the samples were measured by EnviroTest Laboratories (Edmonton, Alberta, Canada) according to U.S. EPA SW-846 method 3050B (U.S EPA 1996). This procedure is summarized in Kwon et al. (2004), along with the results of the As analysis.

### Statistical analysis.

Statistical analyses were performed using SPSS 12.0 (SPSS Inc., Chicago, Illinois, USA) and Microsoft Excel 2003 (Microsoft Corporation, Redmond, Washington, USA). Data are expressed as mean ± SD. Metal concentrations below detection limit (nondetectable) are expressed as half the detection limit of the metal unless otherwise specified in the text. The average concentrations of Cr, Cu, and As in hand-washings of children from CCA playgrounds were compared with those of the children from non-CCA playgrounds via a two-independent-samples *t*-test. The difference in Cr levels between male and female children were also compared via separate two-independent-sample *t*-tests for both CCA and non-CCA playgrounds. A *p*-value < 0.05 was considered statistically significant. Correlation analyses were performed for Cr levels and As levels, as well as for Cr and Cu levels and for As and Cu levels. PCAs were performed for all metal levels analyzed at each playground, using SPSS 12.0. For each playground, all components with eigenvalues > 1 were extracted. The results of the Kaiser-Meyer-Olkin measure of sampling for both CCA (0.7) and non-CCA (0.6) playgrounds were equal to or above the minimum recommended value of 0.6. The correlation matrices in both cases were proven not to be identity matrices by Bartlett’s test of sphericity (*p* < 0.001 in all cases for both CCA and non-CCA playgrounds). Therefore, the hand-washing concentration data meet the minimum standards for PCA. We analyzed 21 components (elements). The relationships between all the variables (i.e., the hand-washing concentrations of all metals examined via ICPMS) were reduced to seven components (components 1–7) for both CCA and non-CCA playgrounds, when nondetectables are assumed as half the detection limit. In this case, in both CCA and non-CCA playgrounds, component 1 accounts for most (30% and 22%) of the variance seen in the data, whereas components 2 and 3 account for approximately 12% (CCA) and 13% (non-CCA), and 11% (CCA) and 10% (non-CCA) of the variance of the data, respectively. If nondetectable levels are assumed as zero, then components 6 and 7, explaining 53% and 45% of the variance of the data, are extracted for CCA and non-CCA playgrounds, respectively. In this case, component 1 accounts for 30% (CCA) and 22% (non-CCA), component 2 for 12% (CCA) and 13% (non-CCA), and component 3 for 11% (CCA) and 10% of the variance. The PCA was based on covariance matrices, as all variables had the same units (parts per billion).

## Results

### Demographics of the participating children.

Compared with the previous study on As (Kwon et al. 2004), three of the samples analyzed previously for CCA playgrounds were not analyzed this time because of spillage from their containers. These samples were for two female children and one male child, ages 4, 2, and 2.5 years, respectively. Exclusion of the three samples does not greatly affect the age distribution of the children (figure not shown). The average ages of the participating children were 4.8 ± 2.5 years for the CCA playground (63 children) and 4.8 ± 2.4 years for the non-CCA playground (64 children). There was no significant difference in age between the two groups (*p* = 0.98). Thus, a total of 127 children (63 from CCA playgrounds and 64 from non-CCA playgrounds) were accounted for in the hand-washing analysis. Sixty-nine (54.3%) of the participating children were boys, and 58 (45.7%) were girls.

### Length of play time.

The mean play time was 76 ± 46 min for CCA playgrounds and 49 ± 28 min for non-CCA playgrounds. The difference between the means is driven by longer play times (> 120 min) for a few children (*n* = 8) in the CCA playgrounds. No correlation was seen between play time and age for children in CCA (*r* = 0.27) or non-CCA (*r* = –0.06) playgrounds. The play times of the children whose samples were spilled were 30, 30, and 60 min. These times are not on the high or low end of the distribution of play time lengths for CCA playgrounds seen in Kwon et al. (2004) and affect its shape minimally.

### Concentration of chromium in the sand/soil from the playgrounds.

Table 1 shows the concentrations of Cr in the sand/soil samples collected from each of the 16 playgrounds. Detection limit was 0.5 mg/kg for Cr and 2.0 mg/kg for Cu. The mean Cr concentrations are 2.0 ± 0.8 (median, 1.8; range, 0.8–4.6) and 1.3 ± 0.4 (median, 1.3; range, 0.7–2.2) mg/kg for CCA and non-CCA playgrounds, respectively. The difference between these concentrations was statistically significant (*p* = 0.003).

The mean concentration of Cu in CCA playgrounds was 2.7 ± 1.8 mg/kg (median, 2.0; range, nondetectable to 8.0). In most non-CCA playgrounds, the concentrations of Cu in the soil were below the detection limit of 2 mg/kg, resulting in a mean value of Cr for all non-CCA playgrounds below detection limit. The difference in Cu concentrations between the CCA and non-CCA playgrounds was also statistically significant (*p* < 0.0005).

### Amount of soluble chromium in the hand-washing samples.

Table 2 summarizes the results of analysis of the hand-washing samples for soluble Cr, insoluble Cr, and total Cr. To determine soluble Cr, the hand-washings were filtered to remove any particulate matter (> 1.2 μm), including sand. Detection limit was 0.01 ng/mL or 2.3 ng for Cr. The concentration of soluble Cr (nanograms per milliliter) is multiplied by the total volume (230 mL) to obtain the number of nanograms Cr on the child’s hands. The overall mean value was 759 ± 575 ng (median, 564; range, nondetectable to 4,761) for CCA playgrounds. For non-CCA playgrounds, the overall mean value was 304 ± 265 ng (median, 272; range, nondetectable to 1,035). The difference between the two means was statistically significant (*p* < 0.003).

### Amount of chromium in the sand residue collected in the hand-washing samples.

The amounts of sand collected from the hand-washing samples, in dry weight, of the sand samples analyzed were 22.0 ± 19.1 mg (median, 16.4 mg; range, 0.8–95.8 mg) for CCA playgrounds and 25.2 ± 23.3 mg (median, 16.6; range, 3.7–116.2 mg) for non-CCA playgrounds (*p* = 0.38). Thus, there was no significant difference between the mean amounts of sand on the children’s hands in CCA and non-CCA playgrounds (Table 2). The mean values of Cr in the sand washed from children’s hands were 409 ± 646 ng (median, 228 ng; range, ND–3,144 ng) in CCA playgrounds and 348 ± 509 ng (median, 164 ng; range, 20–3,147 ng) in non-CCA playgrounds. There was no significant difference between the two groups in sand Cr levels in hand-washings (*p* = 0.56), probably because of the small amounts of sand collected on children’s hands from both CCA and non-CCA playgrounds.

### Total amount of chromium in the hand-washings.

The total amount of Cr (the sum of soluble and insoluble) present in the hand-washing samples is also summarized in Table 2. The mean values were 1,112 ± 1,089 ng (median, 688 ng; range, 78–5,875 ng) for CCA playgrounds and 652 ± 586 ng (median, 492 ng; range, 61–3,377 ng) for non-CCA playgrounds, respectively. The difference in mean total Cr levels was statistically significant between the two types of playgrounds (*p* = 0.004), driven mainly by the soluble Cr. To examine if longer play time (> 120 min) of eight children in the CCA playground could influence the outcome of the comparison, the data set was reanalyzed after removing the data from the eight children who played in the CCA playground for > 120 min. The difference in mean total Cr between CCA and non-CCA playgrounds remained statistically significant (*p* = 0.007), even when measurements from the eight children with long play times were removed from the CCA group. Three samples, two CCA and one non-CCA, were missing in the insoluble Cr analysis because of spillage. The values for mean total Cr in CCA and non-CCA playgrounds do not change if nondetectable Cr levels are taken as zero.

### Correlation of total chromium levels with age, sex, and length of play time.

**Figure 1:**
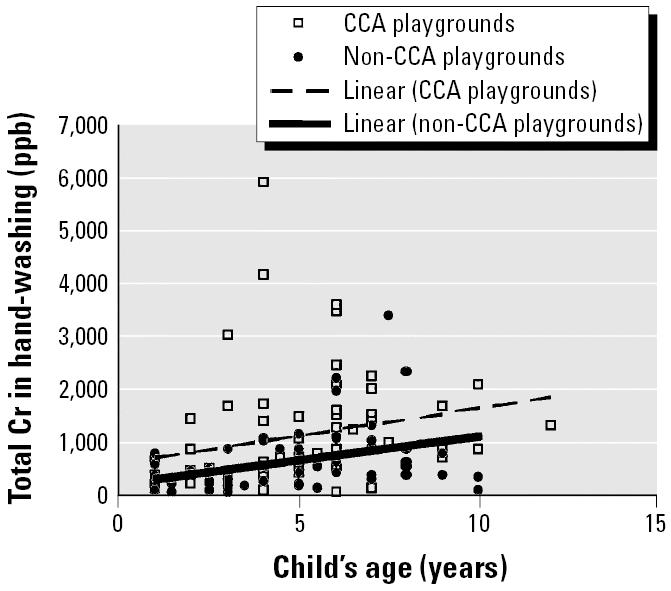
Plot showing absence of correlation between soluble Cr levels in hand-washing samples and children’s age for both CCA and non-CCA playgrounds. Results are for Cr levels (ng) as determined by ICP-MS analysis of the hand-washings of 63 children who played in eight CCA and 64 children who played in eight non-CCA playgrounds. Correlation coefficients are *r* = 0.24 (CCA: *y* = 104*x* + 611) and *r* = 0.35 (non-CCA: *y* = 86*x* + 241).

**Figure 2:**
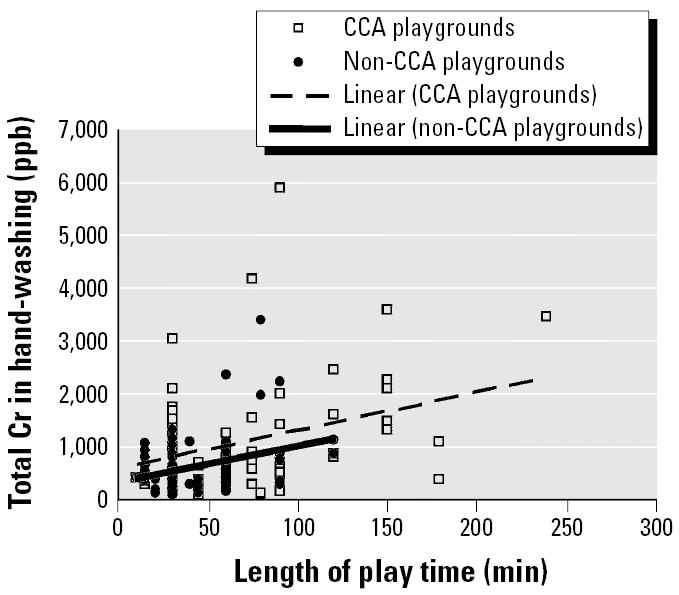
Plot showing absence of correlation of Cr levels in hand-washing samples and length of play time for children in both CCA and non-CCA playgrounds. Results are for Cr levels as determined in Figure 1. Correlation coefficients are *r* = 0.31 (CCA: *y* = 7.3*x* + 560) and *r* = 0.32 (non-CCA: *y* = 6.9*x* + 318).

Figure 1 shows the correlation analysis of total Cr concentrations in hand-washing samples with children’s age. It appears that there is some correlation between the two variables, as the lines have positive slopes, although they are weak correlations for both the CCA (*r* = 0.24) and non-CCA (*r* = 0.35) playgrounds. There is weak (if any) correlation between length of play time and soluble Cr levels in hand-washings of children after playing in CCA (*r* = 0.31) and non-CCA (*r* = 0.32) playgrounds (Figure 2). The conclusions do not change when nondetectable Cr levels are taken as zero. There is weak (if any) correlation between children’s age and levels of Cr (CCA: *r* = 0.24; non-CCA: *r* = 0.35) or between length of play time and levels of Cr (CCA: *r* = 0.31; non-CCA *r* = 0.32) in hand-washing samples.

No statistically significant difference was found between Cr levels in the hand-washings of male and female children, for CCA or non-CCA playgrounds, regardless of whether non-detectable levels of Cr were assumed to be half the detection limit (CCA: *p* = 0.45; non-CCA: *p* = 0.06) or zero (CCA: *p* = 0.45; non-CCA: *p* = 0.23).

### Correlation analysis of soluble chromium, copper, and arsenic levels in hand-washing samples.

To better understand the relationship between Cr, Cu, and As levels on the hands of children after contacting CCA-treated wood, we performed correlation analyses between soluble Cr and Cu, Cr and As, and Cu and As levels in the handwash samples.

**Figure 3:**
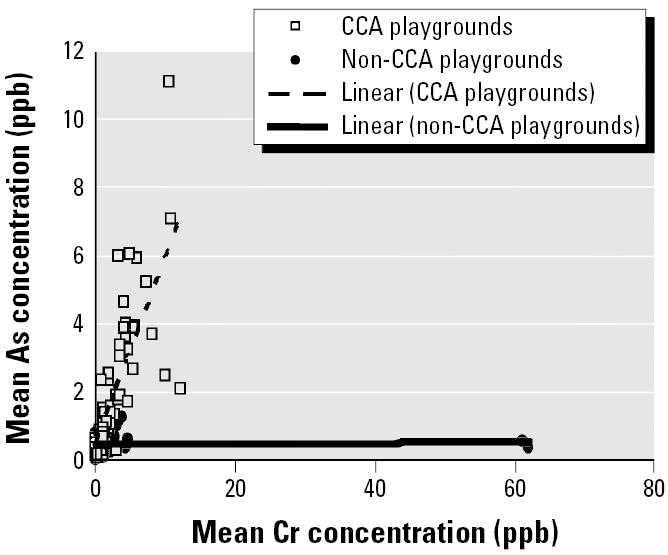
Plot showing strong correlation (*r* = 0.736) between As and Cr levels in hand-washing samples taken from children after playing in CCA playgrounds. Weak correlation was found between As and Cr levels for children in non-CCA playgrounds (*r* = 0.486). Concentration values multiplied by 230 mL would give the amount of Cr (ng).

**Figure 4:**
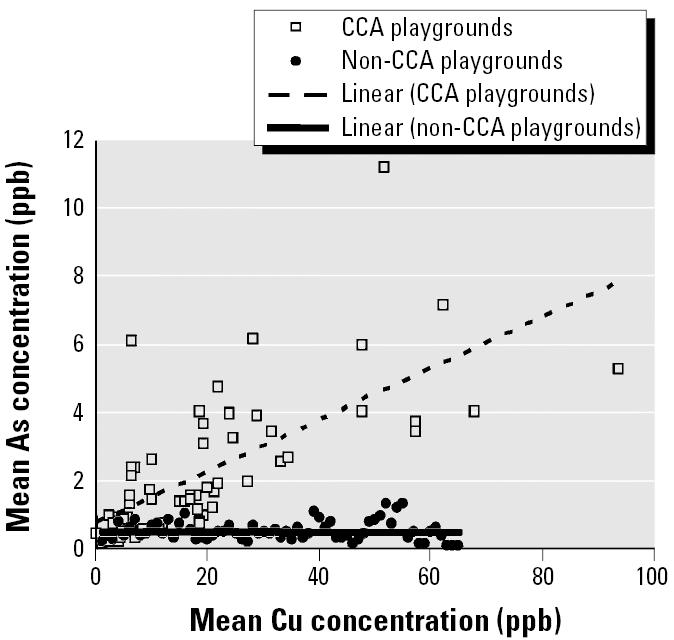
Plot showing strong correlation (*r* = 0.685) between As and Cu levels in hand-washing samples taken from children after playing in CCA playgrounds. Weak correlation was found in non-CCA playgrounds (*r* = 0.503).

Figure 3 shows a strong correlation (*r* = 0.736) between Cr and As levels in samples collected from children playing in CCA playgrounds and a weak correlation in samples from non-CCA children (*r* = 0.486). Similarly, a strong correlation (*r* = 0.782) between As and Cu levels in hand-washings can be seen for CCA playgrounds. However, there is an outlying Cu value (84.2 μg) for CCA playgrounds. Removal of this outlier reduces the strength of this correlation (*r* = 0.685) slightly (Figure 4). The correlation is weaker for non-CCA playgrounds (*r* = 0.503) (Figure 4).

**Figure 5:**
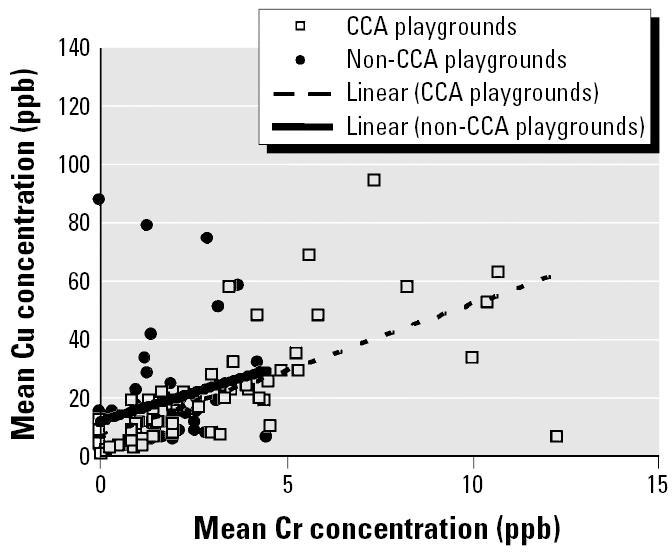
Plot showing strong correlation (*r* = 0.672) between Cr and Cu levels in hand-washings taken from CCA playgrounds. Weak correlation (*r* = 0.252) was seen in non-CCA playgrounds.

A strong correlation (*r* = 0.801) is also seen between Cr and Cu levels in hand-washing samples from CCA playgrounds. Removal of the outlying Cu value (84.2 μg) slightly reduces the correlation (*r* = 0.672). The correlation between Cr and Cu levels in non-CCA samples is much weaker (*r* = 0.252) (Figure 5).

### PCAs on soluble copper, chromium, and arsenic levels.

The relationships between the various concentrations of metals and the first three components are summarized as rotated component loadings (data not shown), which are basically correlations between the variables and the factor patterns (Rummel 1970). A component loading close to an absolute value of one signifies that a variable is highly correlated to a factor pattern, whereas an absolute value close to zero indicates that a variable is not involved in a factor pattern. These loadings are plotted in 3-dimensional rotated space to visualize the metal patterns in CCA and non-CCA hand-washings (figures not shown).

The pattern of metal concentrations is similar between playgrounds. In both cases, there is a main cluster of metals to the right of the component plots, with magnesium, barium, bismuth, thallium, and indium removed from this cluster. As, Cr, and Cu are all in the main cluster. However, they are closer to each other in CCA playgrounds than in non-CCA playgrounds.

None of the metals had consistently high (≥ 0.5) component 2 or 3 loadings except for beryllium, bismuth, indium, thallium, and barium. However, rotated component 1 loading of Cr in CCA playgrounds (0.876) was higher than in non-CCA playgrounds (0.661). Cu component 1 loadings were higher and As component 1 loadings were the same between CCA (As: 0.83; Cu: 0.76) and non-CCA playgrounds (As: 0.78; Cu: 0.48).

## Discussion

The playground sites sampled were matched on location and manufacturer for both CCA and non-CCA playgrounds, and weather conditions during sampling were similar for both types of playground. Sampling days were alternated for both CCA and non-CCA sites. Thus, adequate controls were in place to ensure that any difference in the levels of Cr on children’s hands between CCA and non-CCA playgrounds could be attributed to the type of playground. The length of play time and the age of the children were uncontrolled variables, which maximized the number of children sampled. The distribution of these variables was similar for both CCA and non-CCA playgrounds (Kwon et al. 2004). There was no correlation between age and play time for either type of playground. Statistical tests confirmed that Cr levels found in the hand-wash samples were independent of age (Figure 1) and sex.

The total amount of Cr (Table 2), including both water-soluble Cr in the washing water and insoluble Cr in the sand of the handwash samples, was significantly higher for the CCA group (1,112 ± 1,089 ng) than for the non-CCA group (652 ± 586 ng). This difference was due to the soluble Cr in the handwash water. A statistically significant difference was found between the CCA and non-CCA playgrounds with respect to levels of soluble Cr in the handwash samples (Table 2). The amount of soluble Cr for the CCA group (759 ± 575 ng) was significantly higher than that for the non-CCA group (304 ± 265 ng). This represents an approximately 2-fold difference between the two types of playgrounds. Children 2–6 years of age have frequent hand-to-mouth activity [8–10 contacts per hour, on average (Reed et al. 1999; Tulve et al. 2002)]. Therefore, soluble Cr on their hands could be ingested (Tulve et al. 2002).

The increased levels of Cr found on the hands of the CCA group of children are thought to be rubbed off the CCA-wood by direct contact. A small amount of Cr can be dislodged from CCA-treated wood (Cooper et al. 2001; Hingston et al. 2001; Lebow et al. 2003). It is this dislodgeable Cr that is thought to be transferred to the hands of children when they touch the wood. Figure 2 shows no correlation between play time and total handwash Cr for either CCA (*r* = 0.31) or non-CCA playgrounds (*r* = 0.32). For play times ≤ 30 min, there is weak or no correlation between play time and total handwash Cr for CCA (*r* = 0.51) and non-CCA (*r* = –0.05) playgrounds. This suggests that Cr loading either is saturated at a very early time point or exists in some type of equilibrium between loading and unloading. An absence of correlation was also seen between length of play time and As loading for both CCA (*r* = 0.28) and non-CCA (*r* = 0.33) playgrounds. The results were similar for Cu (CCA *r* = 0.18, non-CCA *r* = –0.28) (data not shown).

A maximum amount of 4.8 μg soluble Cr was found on a child’s hands after play in a CCA playground (Table 2). If an estimated body weight of 17.8 kg (for a child 2–6 years of age) is used, the maximum amount of Cr found is equivalent to 0.3 μg/kg body weight. If it is assumed that all the Cr on a child’s hands is ingested, the measured value is below the maximum estimated average daily intake of total Cr, approximately 1.5 μg/kg and 0.9 μg/kg, for Canadian children in the age ranges 0.5–4 and 5–11 years, respectively [Canadian Environmental Protection Act (CEPA) 1999]. The average daily dietary ingestion of total Cr in Canada is 13.3–16.9 μg for children ages 0.5–4 years, 18.9–21.6 μg for children ages 5–11 years, and 27.3 μg for adults (CEPA 1999). The average daily dietary ingestion of total Cr for an adult is 25–224 μg in the United States (ATSDR 2000), 100 μg in Spain (Garcia et al. 2001), 59.9 μg in India, and 224 μg in Japan (Iyengar et al. 2002). A chronic oral reference dose of 3 μg/kg/day is given for ingestion of Cr(VI) (U.S EPA 1998). The threshold concentration of Cr(VI) for skin hypersensitivity is 10 mg/kg body weight (ATSDR 2000; Bagdon and Hazen 1991).

The 2-fold difference in the amount of soluble Cr on children’s hands between CCA and non-CCA playgrounds is not as great as that previously found for As. The difference in As amounts between CCA and non-CCA playgrounds was 5-fold (Kwon et al. 2004). This is consistent with other studies showing that As is more readily dislodged from CCA wood than Cr (Fahlstrom et al. 1967; Henshaw 1979; Hingston et al. 2001).

Cr in CCA wood is present primarily in insoluble Cr(III) forms complexed to the wood lignin, cellulose, and carbohydrates (Hingston et al. 2001; Pizzi 1990a, b). Cr–As complexes can also bind wood components (Bull 2001; Lebow et al. 2004b). Any soluble Cr [Cr(VI)] present after wood treatment is fixed (reduced to the trivalent form) with time (Bull 2000, 2001; Henshaw 1979; Nico et al. 2004). At 98.2% Cr fixation, a small fraction (2 μg/cm^2^) of total Cr, Cu, or As is leached (Hingston et al. 2001). However, this fixation process is slow, and Cr(VI) can remain in CCA wood for over 6 months (Bull 2001). In addition, Cr(III) is considered leachable when it undergoes ligand exchange reactions, thus detaching from the wood components and precipitating onto the wood surface, where it is weakly absorbed (Bull 2000, 2001; Nico et al. 2004). The exact form in which Cr is leached is unclear, but it has been suggested to be leachable by itself as Cr(VI) and in the form of various Cr arsenates (Pizzi 1990a, 1990b). Results of correlation analyses found a strong correlation between Cr and As levels on children’s hands after contacting CCA wood (*r* = 0.736; Figure 3). This correlation is absent in non-CCA playgrounds (*r* = 0.486; Figure 3).

The form in which Cu is leached from CCA wood is unknown, but Cu arsenates and some form of Cu–Cr–As complexes (Hingston et al. 2001) have been suggested as possible leachable species (Lebow et al. 2003). Another possibility is that Cu leaches independently of Cr and As as a result of binding different spaces on the wood (Bull 2000, 2001). A strong correlation was present between Cu and As levels in hand-washings from CCA (*r* = 0.685) but not non-CCA (*r* = 0.503) playgrounds (Figure 4). Likewise, a strong correlation was present between Cu and Cr in CCA (*r* = 0.672) but not non-CCA (*r* = 0.252) playgrounds (Figure 5). Thus, these results (Figures 3–5) suggest that Cu, Cr, and As co-leach from CCA-treated wood, either as dislodgeable complexes/residues or as separate species.

Further, PCA was carried out to determine whether there is a characteristic pattern of Cu, Cr, and As in CCA hand-washings different from that in non-CCA hand-washings. When a nondetectable level of a metal is taken as half of its ICPMS detection limit, seven components are extracted, accounting for approximately 70% (non-CCA) and 77% (CCA) of total data variation. Components 1, 2, and 3 are used to create rotated component plots (figures not shown), which summarize the data in pattern form. The bulk of the metals, including As, Cu, and Cr, are grouped in a main cluster and are correlated primarily to component 1. Beryllium, bismuth, indium, thallium, barium, and cadmium are grouped separately from the other metals, with stronger correlations to components 2 and 3. Their presence in the hand-washings is probably not a result of contact with CCA-treated wood, but of contact with other substances in the environment. The correlations of As, Cr, and Cu levels to component 1 are essentially higher in CCA and non-CCA playgrounds. Thus, it seems that hand-washing samples from CCA playgrounds show grouping patterns of CCA components absent in non-CCA samples.

Kwon et al. (2004) found no significant difference in the amount of As in sand between CCA and non-CCA playgrounds. However, this is not the case for Cr. Analysis of sand/soil samples from the playgrounds for Cr levels shows a statistically significant difference between CCA and non-CCA playgrounds. Previous studies have shown Cr leaching into soil adjacent to CCA-treated poles, decks, and structures (Balasoiu et al. 2001; Chirenje et al. 2003; Cooper et al. 2001). However, the increased levels of soluble Cr seen on the hands of children in the CCA group, as well as the stronger correlation of Cr levels with the CCA playground pattern, are likely due to increased Cr exposure via direct contact with CCA-treated wood, because insoluble Cr levels in the hand-washings are not significantly different between CCA and non-CCA playgrounds. The concentrations of soil/sand Cr for each type of playground (CCA: 2.0 ± 0.8 mg/kg; non-CCA: 1.3 ± 0.4 mg/kg) are above the Canadian guideline level (0.4 mg/kg) for hexavalent Cr in all land use (residential/parkland) in Canada [Canadian Council Ministry of the Environment (CCME) 1995]. However, soil and sand Cr is predominantly present in the insoluble Cr(III) form (for which there is no CCME guideline level (CCME 1995), and thus is unlikely to enter the hand-washing samples (U.S. EPA 1998).

Cr is ubiquitous in the natural environment. The toxicity of Cr varies dramatically, depending on its speciation. Although Cr(III) in small amounts is an essential nutrient, Cr(VI) is highly toxic. Because Cr(VI) is water soluble, we recommend that children wash their hands after playing on CCA-treated wood.

## Conclusions

Children have approximately two times more Cr on their hands after playing in playgrounds containing CCA-treated wood structures than non-CCA playgrounds. This increased level of Cr is probably due to direct contact with CCA-treated wood and subsequent transfer of Cr and Cr complexes onto children’s hands. It could also be due to direct contact with sand adjacent to surrounding CCA-treated structures, which is less likely, as most Cr in soil/sand is insoluble. Soluble Cr was washed off the children’s hands with water and into the hand-washing samples. The maximum amount of Cr found on children’s hands was 5.9 μg (4.8 μg soluble), which is much lower than the average daily intake of total Cr in the Canadian diet (13–27 μg).

Correlation analyses indicate strong associations between Cr, Cu, and As levels in CCA hand-washings that are absent in non-CCA hand-washings. PCA provides further evidence that Cr, As, and Cu in hand-washing samples from children who played in CCA playgrounds are grouped. These results point to the co-leaching of these three elements from CCA wood.

## Correction

Some of the values in the section “Amount of chromium in the sand residue collected in the hand-washing samples” were incorrect in the original manuscript published online. They have been corrected here.

## Figures and Tables

**Table 1:** Cr concentrations (mg/kg) in sand/soil samples collected from 16 playgrounds.

Playground	Mean ± SD	Median	Range
CCA playgrounds			
A	2.7 ± 0.9	2.6	1.9–3.6
C	1.6 ± 0.5	1.3	1.3–2.1
D	2.7 ± 1.3	2.6	1.5–4.0
F	2.5 ± 1.8	1.8	1.2–4.6
G	2.2 ± 0.7	2.1	1.3–3.7
I	2.4 ± 0.7	2.3	1.8–3.1
N	1.5 ± 0.7	1.1	1.0–2.3
R	1.6 ± 0.4	1.6	0.8–2.4
Overall	2.0 ± 0.8	1.8	0.8–4.6
Non-CCA playgrounds			
B	1.1 ± 0.2	1.0	0.9–1.3
E	1.1 ± 0.3	1.0	0.9–1.4
H	1.1 ± 0.4	1.0	0.7–1.5
J	1.5 ± 0.3	1.5	1.2–1.7
K	1.5 ± 0.4	1.3	1.2–1.9
L	1.3 ± 0.1	1.3	1.3–1.4
M	1.7 ± 0.5	1.8	1.2–2.2
O	1.4 ± 0.3	1.6	1.1–1.6
Overall	1.3 ± 0.4	1.3	0.7–2.2

**Table 2:** Water soluble Cr, insoluble Cr in sand, and total Cr in hand-washings from children playing in eight CCA and eight non-CCA playgrounds.

	Soluble Cr (ng)			Sand on hands (mg)			Insoluble Cr (ng)			Total Cr (ng)		
Playgrounds	Mean ± SD	Median	Range	Mean ± SD	Median	Range	Mean ± SD	Median	Range	Mean ± SD	Median	Range
CCA
A	821 ± 690	690	230–2,300	24.2 ± 24.9	13.9	5.0–77.7	223 ± 157	146	34–500	1,044 ± 703	836	264–2,426
C	202 ± 184	230	ND–460	21.9 ± 23.0	14.0	5.2–76.5	541 ± 1,076	132	37–2976	772 ± 1,186	406	134–3,436
D	1,557 ± 1,219	1,219	736–4,761	26.7 ± 10.3	26.3	15.0–42.8	568 ± 338	427	366–1,128	1,728 ± 373	1,705	1,278–2,246
F	334 ± 7	345	322–345	20.4 ± 21.8	20.4	5.0–35.8	27 ± 30	27	6.0–48	360 ± 37	360	334–386
G	920 ± 644	644	207–2,461	31.7 ± 20.4	29.5	3.3–67.7	724 ± 949	454	ND–3,144	1,647 ± 1,222	1,503	405–4,138
I	506 ± 322	483	184–1,219	18.9 ± 11.1	14.5	6.6–38.2	221 ± 127	188	56–395	730 ± 384	672	253–1,577
N	299 ± 276	230	ND–1,058	11.4 ± 10.1	7.7	0.8–38.3	158 ± 129	120	ND–337	469 ± 382	365	78–1,392
R	1,426 ± 1,104	1,334	230–2,829	29.7 ± 31.0	16.4	10.8–95.8	768 ± 1,075	315	97–3,056	2,284 ± 2,098	1,615	173–5,875
Overall	759 ± 575	564	ND–4,761	22.0 ± 19.1	16.4	0.8–95.8	409 ± 646	228	ND–3,144	1,112 ± 1,089	688	78–5,875
Non–CCA
B	230 ± 115	230	ND–460	40.5 ± 40.3	28.7	7.2–116.2	720 ± 1,038	235	110–3,147	950 ± 1,038	465	340–3,377
E	391 ± 92	437	276–506	21.3 ± 13.2	21.2	6.5–37.3	195 ± 146	166	42–384	590 ± 219	562	338–856
H	322 ± 161	322	184–598	15.1 ± 9.2	13.0	5.7–38.2	144 ± 148	81	36–552	466 ± 265	387	107–1,094
J	598 ± 299	598	184–1,035	27.5 ± 32.9	13.3	9.1–86.1	439 ± 446	395	39–1,177	1,046 ± 732	1,037	211–2,203
K	69 ± 207	ND	ND–626	25.3 ± 10.8	24.8	10.2–45.7	343 ± 278	236	60–859	405 ± 306	268	61–860
L	331 ± 345	299	ND–989	9.0 ± 2.9	9.5	3.7–11.7	89 ± 45	88	20–161	419 ± 347	356	92–1,054
M	621 ± 161	598	460–851	38.2 ± 23.9	45.3	10.8–73.1	480 ± 616	178	60–1,815	1,106 ± 546	896	778–2,322
O	138 ± 184	46	ND–299	23.8 ± 22.6	15.3	3.8–70.2	356 ± 347	228	65–1,045	494 ± 482	326	66–1,284
Overall	304 ± 265	272	ND–1,035	25.2 ± 23.3	16.6	3.7–116.2	348 ± 509	164	20–3,147	652 ± 586	492	61–3,377

ND, nondetectable.
